# Effectiveness of artificial intelligence screening in preventing vision loss from diabetes: a policy model

**DOI:** 10.1038/s41746-023-00785-z

**Published:** 2023-03-27

**Authors:** Roomasa Channa, Risa M. Wolf, Michael D. Abràmoff, Harold P. Lehmann

**Affiliations:** 1 Department of Ophthalmology and Visual Sciences, University of Wisconsin, Madison, WI USA; 2 Department of Pediatrics, Division of Endocrinology, Johns Hopkins Medicine, Baltimore, MD USA; 3 Department of Ophthalmology and Visual Sciences, University of Iowa, Iowa City, IA USA; 4 Department of Medicine, Section on Biomedical Informatics and Data Science, Johns Hopkins University, Baltimore, MD USA

**Keywords:** Computational biology and bioinformatics, Systems biology

## Abstract

The effectiveness of using artificial intelligence (AI) systems to perform diabetic retinal exams (‘screening’) on preventing vision loss is not known. We designed the Care Process for Preventing Vision Loss from Diabetes (CAREVL), as a Markov model to compare the effectiveness of point-of-care autonomous AI-based screening with in-office clinical exam by an eye care provider (ECP), on preventing vision loss among patients with diabetes. The estimated incidence of vision loss at 5 years was 1535 per 100,000 in the AI-screened group compared to 1625 per 100,000 in the ECP group, leading to a modelled risk difference of 90 per 100,000. The base-case CAREVL model estimated that an autonomous AI-based screening strategy would result in 27,000 fewer Americans with vision loss at 5 years compared with ECP. Vision loss at 5 years remained lower in the AI-screened group compared to the ECP group, in a wide range of parameters including optimistic estimates biased toward ECP. Real-world modifiable factors associated with processes of care could further increase its effectiveness. Of these factors, increased adherence with treatment was estimated to have the greatest impact.

## Introduction

Digital Technology, including autonomous Artificial Intelligence (AI), has the potential to improve patient outcomes, reduce health disparities, improve access to care, and lower health-care costs^1–5^. Typical metrics for evaluation of new technology focus on efficacy^6,7^. In the case of diagnostic AI systems these efficacy metrics translate into diagnostic-accuracy measures, such as sensitivity and specificity, compared to an agreed upon reference standard^8^. Multiple AI systems have been shown to be safe and efficacious using such metrics, resulting in FDA De Novo clearance and clinical use^9–11^. While these diagnostic-accuracy metrics correctly estimate the efficacy of the diagnostic AI system, they do not give information on the overall impact (effectiveness) of the AI system on patient outcomes^6^.

Instead, the impact of implementing AI on patient outcome is dependent on many factors beyond the diagnostic accuracy of AI^7^. These factors include characteristics of the disease, such as prevalence, and natural history, as well as potential frictions in the care process, including access to care, adherence with a recommended referral, and adherence with treatment and management recommendations. In addition, treatment itself where indicated is unlikely to be perfect, and may itself lead to imperfect outcomes. Therefore, even if a diagnostic AI with perfect accuracy is implemented, outcomes will be affected by these frictions associated with processes of care, as will potential efficiency gains, and differential effects on health inequities. These processes of care frictions/imperfections may be less obvious, as they cannot be determined from inspection of the standalone AI system, but instead depend greatly on how the AI system is integrated into the care process as well as the health delivery network. While some AI systems, such as those used in the critical care environment may affect patient outcome in real time, in many cases, AI systems are designed for chronic conditions, where a difference in outcome may take years or even decades to manifest. Thus, process-of-care metrics need to be considered in addition to outcomes to determine whether it is worth designing, developing, validating, implementing, regulating and reimbursing such AI systems^6^.

An example of an AI system that has the potential to affect real-world outcomes is the first diagnostic autonomous AI (IDx-DR, Digital Diagnostics Inc, Coralville, Iowa). It received US FDA De Novo clearance in 2018 to autonomously, that is without human oversight, diagnose diabetic retinopathy and macular edema—Diabetic Retinal Disease (DRD)^12^. Clearance was based on efficacy, as determined in a preregistered clinical trial^11^, which provided information on the diagnostic-accuracy metrics of sensitivity and specificity, but not on effectiveness or impact on patient outcomes. As diabetes is a chronic disease, it will take years to determine this impact, requiring following each patient that interacted with the AI system to a disease endpoint for years. Given the lack of such empirical data of the impact on patient outcome (vision loss), we modeled screening strategies and the downstream care process, as the Care Process for Preventing Vision Loss from Diabetes (CAREVL) policy model, to estimate the impact on patient outcome (vision loss).

The primary purpose of this study was to develop the CAREVL model and leverage it to determine the differential impact of autonomous AI-based diabetic retinal exams (‘screening’) vs screening performed in the clinic by an eye care provider (ECP). Secondarily, its purpose was to explore how processes of care modulate the effectiveness of screening strategies.

## Results

All analytical inputs are listed in Table 1 and detailed in the Supplementary.

**Table 1:** Parameters for the decision model.

Parameter Description	Base-case estimate	For sensitivity analysis	
		Low	High
*Population-metrics: Prevalence and natural history of disease*			
Prevalence of Metabolic DRD^11^	0.22	0	0.40
Prevalence of Ophthalmic DRD^11^	0.0088^a^	0	0.10
Prevalence of DRD with Vision Loss^42,43^	0.01	0	0.05
No DRD to metabolic DRD^44^	0.05	0	0.15
Metabolic DRD to ophthalmic DRD^44^	0.02	0	0.20
Ophthalmic DRD to vision loss^39,40^	0.075	0	0.20
Vision loss to irreversible vision loss^45^	0.37	0	0.50
*Diagnostic-accuracy metrics: Sensitivity and Specificity of Screening Strategies*			
Sensitivity of screening for DRD with AI^11,13,17^	0.87	0	1
Sensitivity of screening for DRD with ECP^46^	0.33	0	1
Specificity of screening for DRD with AI^11,13,17^	0.91	0	1
Specificity of screening for DRD with ECP^46^	0.99	0	1
*Process-of-care metrics: Screening and Referral for Appropriate Care*			
Probability that patient follows up for eye care after AI screen positive^4,47–49^	0.75	0	0.95
Probability that patient follows up for eye exams after ECP screen positive^47^	0.29	0	1
Probability of patient Accepting Screening by AI^50^	0.95	0	1
Probability of patient Accepting Screening by ECP^4,5,27,47,51,52^	0.20	0	0.80
Probability that patient with Vision Loss Accepts referral to ECP^28^	0.58	0	0.75
*Process-of-care metrics: Effectiveness of treatments for DRD(Progression of treated disease)*			
Metabolic DRD to ophthalmic DRD^44^	0.01	0	0.05
Ophthalmic DRD to vision loss^39,41,53^	0.02	0	0.50
Vision loss to irreversible vision loss^54^	0.034	0	0.05
*Process-of-care metrics: Probability of Adherence to Treatment*			
Adhering to metabolic management^24,25,55^	0.24	0	1
Adhering to ophthalmic management^26,28^	0.26^a^	0	1
Adhering to DRD vision loss management^28^	0.41^a^	0	1

^a^Calculated values: see Supplementary.

### Base-case and sensitivity analysis

For the base case, in the no-screening strategy the proportion of adults with DM who are estimated to develop any vision loss at 5 years is 1637/100,000; it is 1625/100,000 for the ECP screening strategy, and 1535/100,000 for the AI screening strategy. Thus, the proportion of DM participants who develop vision loss in the model with AI-based screening (1535/100,000) is estimated to be 102/100,000 lower compared with no-screening (1535/100,000) and 90/100,000 lower compared with ECP-based screening (1625/100,000). The difference between no-screening and ECP is 12/100,000. Thus, CAREVL suggests that introduction of an AI-based screening strategy is 8.6 times more effective at preventing vision loss than ECP, under base-case assumptions. No meaningful thresholds were found in one-way or two-way sensitivity analyses (See Table 2, supplementary Table 1 and supplementary Fig. 2 in Supplementary). The results of the two-way sensitivity analysis (supplementary Table 1) showed that across the broad range of sensitivity values AI dominated over ECP across all ranges for the following two-way comparisons: sensitivity and specificity of AI vs ECP screening, and accepting AI vs ECP screening. For the comparison regarding accepting referral after AI vs ECP screening, AI dominated except in the unlikely scenario of low probability of accepting referral after AI and a high probability of accepting referral after ECP. This scenario is far from the base-case, for further clarification, the output of this two-sensitivity analysis is shown in supplemental Fig. 3. In 2019, an estimated 37.3 million Americans had diabetes. If we use a conservative estimate of 30 million Americans with diabetes, based on the numbers above we anticipate that AI-based screening strategy is expected to prevent vision loss in over 27,000 more Americans at 5 years as compared to ECP-based strategy, under base-case assumptions.

**Table 2:** One-way sensitivity analyses.

Population, diagnostic-accuracy, and process-of-care metrics	Type of metric	Base Case (minimum, maximum value for sensitivity analysis)^a^		Threshold^d^
		ECP	AI	
Prevalence of Metabolic DRD	Population	0.22 (0, 0.4)	0.22 (0,0.4)	AI dominates
Offered and accepts screening for DRD	Process of care	0.2 (0,0.8)	0.95 (0,1)	AI dominates
Sensitivity of the DRD screening strategy	Diagnostic accuracy	0.33 (0,1)	0.87 (0,1)	AI dominates
Specificity of DRD screening strategy	Diagnostic accuracy	0.99 (0,1)	0.91 (0,1)	AI dominates
Accepts referral for eye care after a positive screen	Process of care	0.29 (0,1)	0.75 (0, 0.95)	0.08^b^
Probability of adhering with treatment	Process of care	0.24 (0,1)	0.24 (0,1)	AI dominates
Effectiveness of ophthalmic treatments	Process of care	0.02 (0,0.5)	0.02 (0,0.5)	0.1^c^
Effectiveness of metabolic treatments	Process of care	0.01 (0,0.05)	0.01 (0,0.05)	AI dominates

*DRD* diabetic retinal disease, *ECP* eye care provider, *AI* artificial intelligence.

^a^The no-screening option is dominated by AI or ECP in all scenarios and has therefore not been included in this table.

^b^Far from base case value of 0.75.

^c^ Far from base case value of 0.02.

^d^AI dominates refers to the finding that AI is the preferred strategy on each of the one-way sensitivity analyses (across the range of the minimum and maximum values for the parameter specified in parenthesis next to the base-case value).

### Maximal scenarios

**Fig. 1:**
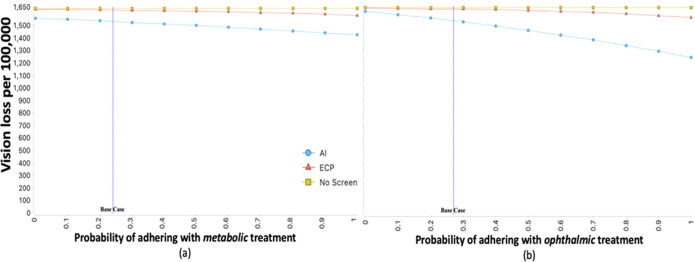
Expected vision loss per 100,000 vs probability of adhering with treatment for each screening strategy. **a**, **b** Show that as the adherence with recommended metabolic and ophthalmic treatments increases the number of patients with vision loss per 100,000 decreases for both the eye care provider (ECP) and artificial intelligence (AI) screening strategies. However, the decrease in number with vision loss is more marked for the AI vs ECP screening strategy.

**Fig. 2:**
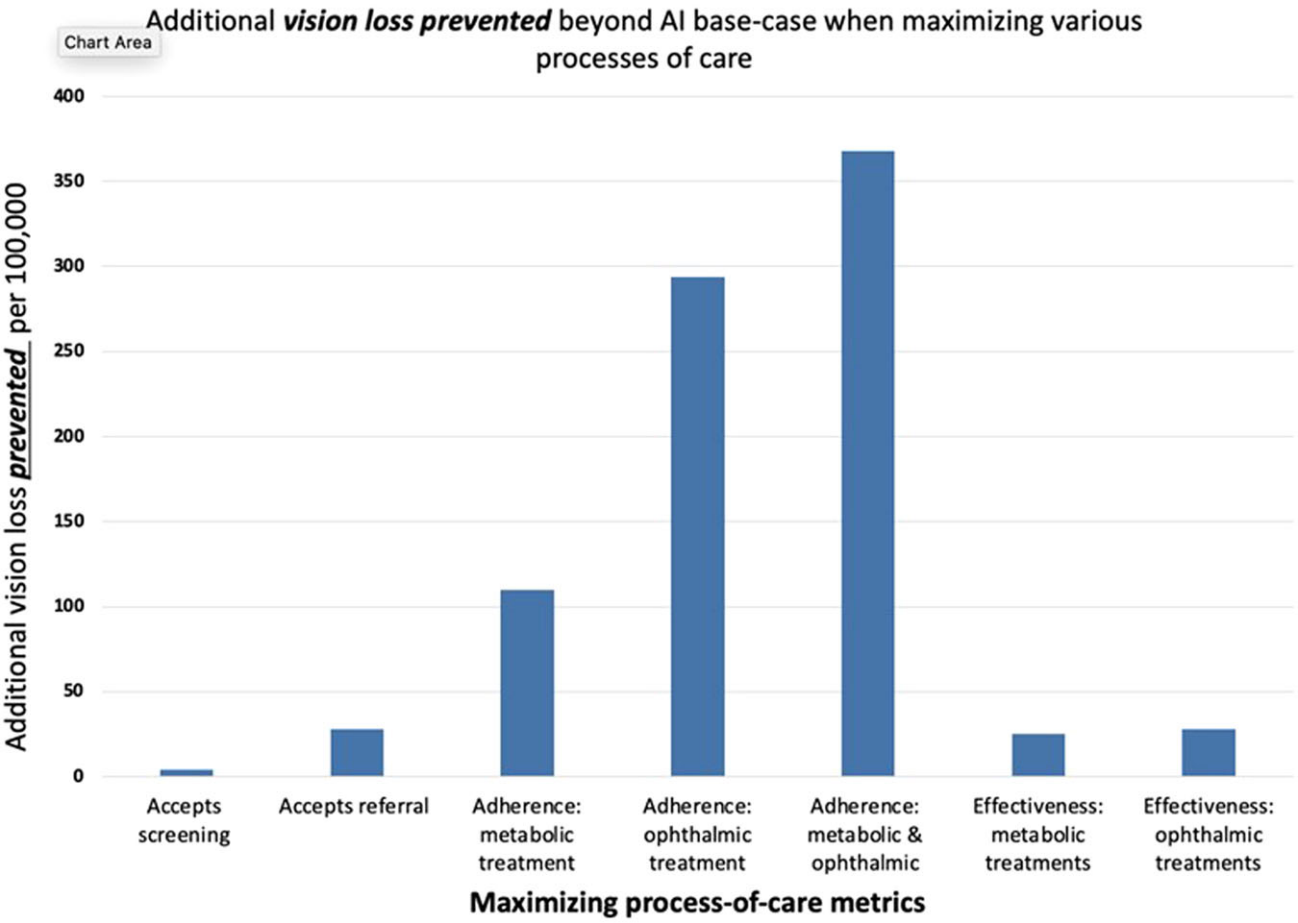
Additional vision loss prevented beyond AI base-case when maximizing processes of care. Figure 2 shows the additional impact on vision loss prevented beyond the base-case scenario when each of the processes of care are maximized. The largest impact on vision loss is estimated to be from maximizing adherence with ophthalmic treatment, followed by adherence with metabolic treatments. Maximizing effectiveness of current metabolic and ophthalmic treatments has a lower impact.

The scenario analyses show that, if adherence to recommended metabolic and ophthalmic treatment were maximized, the estimated total number with any vision loss at 5 years would be lower for both AI and ECP strategies when compared to the base case, with a higher reduction noted for the AI strategy. In the scenario that maximizes adherence to recommended treatment, the number with any vision loss by 5 years is estimated to be 1167/100,000 for the AI strategy, an additional reduction of 367/100,000 from the AI base case. In this scenario, the estimated total number with any vision loss for the ECP strategy instead is 1488/100,000, an additional reduction of 137/100,000 from the ECP base case. In all scenarios tested, the number with vision loss per 100,000 is lower with the AI strategy compared with the ECP strategy. Figure 1 shows the relative impact of increasing the probability of adhering with metabolic and ophthalmic treatments on projected vision loss for each screening strategy. Figure 2 shows the impact of maximizing diagnostic and process-of-care metrics on vision loss when using the AI screening strategy. The largest impacts are when adherence with recommended metabolic or ophthalmic treatments is maximized. The scenario with maximum adherence to metabolic treatment (100%) results in 110/100,000 fewer patients progressing to vision loss. The scenario with maximum adherence to ophthalmic treatment (100%), results in 294/100,000, fewer patients progressing, and maximizing both results in 367/100,000 fewer patients progressing. These numbers suggest an accretive effect of adherence to both metabolic and ophthalmic treatment. Using a conservative estimate of 30 million Americans with diabetes this translates into vision loss prevented in over 110,000 additional Americans with diabetes when AI-based screening is introduced, and treatment adherence is maximized. Maximizing the effectiveness of metabolic and ophthalmic treatments themselves, namely more effective drugs or procedures—does have a marginal impact of 25–28/100,000 fewer progressing, but this is only 6.8–7.6% of the benefit achieved by maximizing adherence to therapies that are currently available.

## Discussion

Using CAREVL, we conclude that autonomous AI is expected to be more effective than ECP-based screening at preventing vision loss among patients with diabetes. This effectiveness can be maximized by improving processes of care, particularly adherence with recommended treatments. Under base-case assumptions, introducing AI in a no-DRD-screening scenario is estimated to be 8.6 times more effective at preventing vision loss from DRD compared with introducing ECP-based screening. The expected differences between ECP and autonomous AI screening strategies are likely due to a combination of factors. While the *efficacy* of AI screening systems in detecting referrable DRD has been established in prospective trials^11,13^, the main drivers of higher *effectiveness* are likely the point-of-care availability of AI and immediate diagnostic output which make it more likely for a patient to accept screening and the recommended referral^4,14^.

The CAREVL model is novel in that it allows evaluating the effectiveness of AI algorithms within the context of real-world patient workflow and the health-care system. Our approach is based on standards for performing and reporting modeling of expected impact of digital health technologies^7,15,16^.Studies evaluating AI have traditionally focused on its diagnostic-accuracy metrics for a given task^11,17–19^. However, as we have shown, to evaluate the effectiveness of AI in the real-world we need to know not only its diagnostic accuracy or how it performs in controlled research settings, but its impact on patient outcomes^7^. This is important because many digital and non-digital health interventions may work well in an ideal ‘model’ setting, but real-world evaluation often reveals outcomes that are much less compelling compared to what can be achieved in a clinical trial setting^6,20–22^.

The CAREVL model further allows us to study the expected impact of adjusting process-of-care metrics on patient outcomes and to identify which metrics may be most important in maximizing the effectiveness of AI within a health-care system, given a chosen strategy. This effort has immediate real-world implications for the implementation of AI-enabled patient-centered care. The CAREVL model suggests that the full potential of AI algorithms in preventing vision loss can be achieved by optimizing processes of care. Among the process-of-care metrics evaluated in the model, adherence with recommended metabolic and ophthalmic treatments had the largest impact on preventing vision loss. Prior studies such as the one by Rohan, et al., have estimated that screening and early treatment of DRD can prevent vision loss and reduce risk of blindness by an estimated 56%^23^. However, this estimate is predicated on perfect-world assumptions of 100% of patients accepting screening, high sensitivity of detecting referrable disease (88%), and 100% complying with recommended treatments. Real-world data from the US regarding adherence to metabolic management show that, on average, only about 22% of patients with diabetes achieve the recommended lipid, blood pressure and glucose control and only about 24% of patients with Type 2 DM achieve a glycated-hemoglobin level of <8%^24,25^. Adherence with recommended screening eye exams for DRD and follow-up eye care is similarly low^26^. Analysis of insured patients with diabetes showed that only about 15%^27^ met the American Diabetes Association’s recommendation for annual DRD screening and data from the National Health Interview Survey showed that only about a third of insured adults in the US followed up for eye care in the absence of visual impairment^28^. These low rates are concerning, as DRD is asymptomatic until late stages, hence the existence of Healthcare Effectiveness Data and Information Set (HEDIS) and Merit-based Incentive Payment System quality measures that incentivize diabetic retinal exams to be performed early and regularly^29^.

The CAREVL model confirms that improving adherence with both the current metabolic and ophthalmic treatments is key to maximizing the success of implementing DRD screening strategies. The model suggests that when autonomous AI is used as a screening strategy, maximizing adherence with metabolic and ophthalmic treatments prevents vision loss in an additional 367 patients/100,000. This reduction is ~4 times more than just introducing AI without improving the process of care. While it remains important to develop increasingly effective treatments for metabolic and ophthalmic DRD, CAREVL suggests that its population impact on vision loss is much lower (~one-tenth) than that of maximizing adherence with existing treatments. This projected impact has important clinical and public health implications. Diabetes is a chronic disease that currently affects almost 37 million adults in the U.S^30,31^, thus introducing AI-based screening could potentially prevent vision loss in an additional 27,000 patients with diabetes over the current ECP-based standard of care. Introducing AI and optimizing processes of care, particularly adherence with recommended treatment, could potentially prevent vision loss in an additional 110,000 patients. These benefits are expected to accrue as the prevalence of diabetes continues to rise. A more nuanced estimate would require further modeling to account for age distributions, annual incidence of diabetes and patient mortality.

The strength of our study is that we developed a real-world model, CAREVL, defining how to evaluate the effectiveness of an AI-based technology on patient outcomes. CAREVL is a relatively novel and more patient-centered approach to evaluating AI technologies as opposed to the overwhelming focus on evaluating diagnostic accuracy. Evaluating the impact of AI and digital health technologies on patient outcomes is an evolving area of research and we have made the model publicly available and invite others to contribute to it. The CAREVL model and this study have limitations. We relied on available, published and peer-reviewed data for the various metrics. It is important to collect real-world data over time, particularly with regards to ECP parameters, to further validate this model. This model does not address costs or utilities. As we are focused on effectiveness, we have considered vision loss in either eye. In future analyses focused on cost and disability benefits it may be better to consider vision loss in the worse seeing eye^32,33^. We did not compare the effectiveness of AI-based screening strategies with telemedicine programs as there is considerable variation between programs but once the relevant metrics identified in the model are collected, the relative effectiveness of telemedicine programs can be determined. One of the limitations of the study is that we did not model the benefit of ophthalmic encounters with an ECP as opposed to AI in potentially detecting diseases other than DRD (e.g., cataracts, macular degeneration, glaucoma). Our rationales for this decision were that (a) patients with visually significant cataract will have vision impairment (by definition) and will visit eye care instead of entering the screening pathway; (b) the number of potential missed cases is small: the pivotal trial that led to FDA approval of autonomous AI estimated that 0.2% of participants with glaucoma and 1.6% with non-exudative age-related macular degeneration may be missed by AI-based screening, no cases of neovascular age-related macular degeneration were noted^11^. Furthermore, the United States Preventive Services Task Force has determined that there is insufficient evidence to recommend screening for impaired vision from age-related changes such as age-related macular degeneration and glaucoma at this point^34,35^. We have analyzed the overall impact of autonomous AI in the US, and not the outcomes of specific sub-groups within a population—these require more sophisticated models that we are currently developing. Nevertheless, we expect that CAREVL can be extended in well-established ways to help in answering questions regarding the impact of new technology on real-world outcomes from multiple perspectives (healthcare system, payor, or society).

In summary, our novel CAREVL model suggests that AI-based DRD screening is more effective at preventing vision loss from diabetes than ECP-based screening, and that this effectiveness can be further enhanced by optimizing processes of care. As use of digital health technology and AI increases in the health-care system, this comprehensive model may serve as a framework for evaluating and estimating the real-world impact of digital technologies on patient outcomes in other chronic disease scenarios.

## Methods

### Model structure

**Fig. 3:**
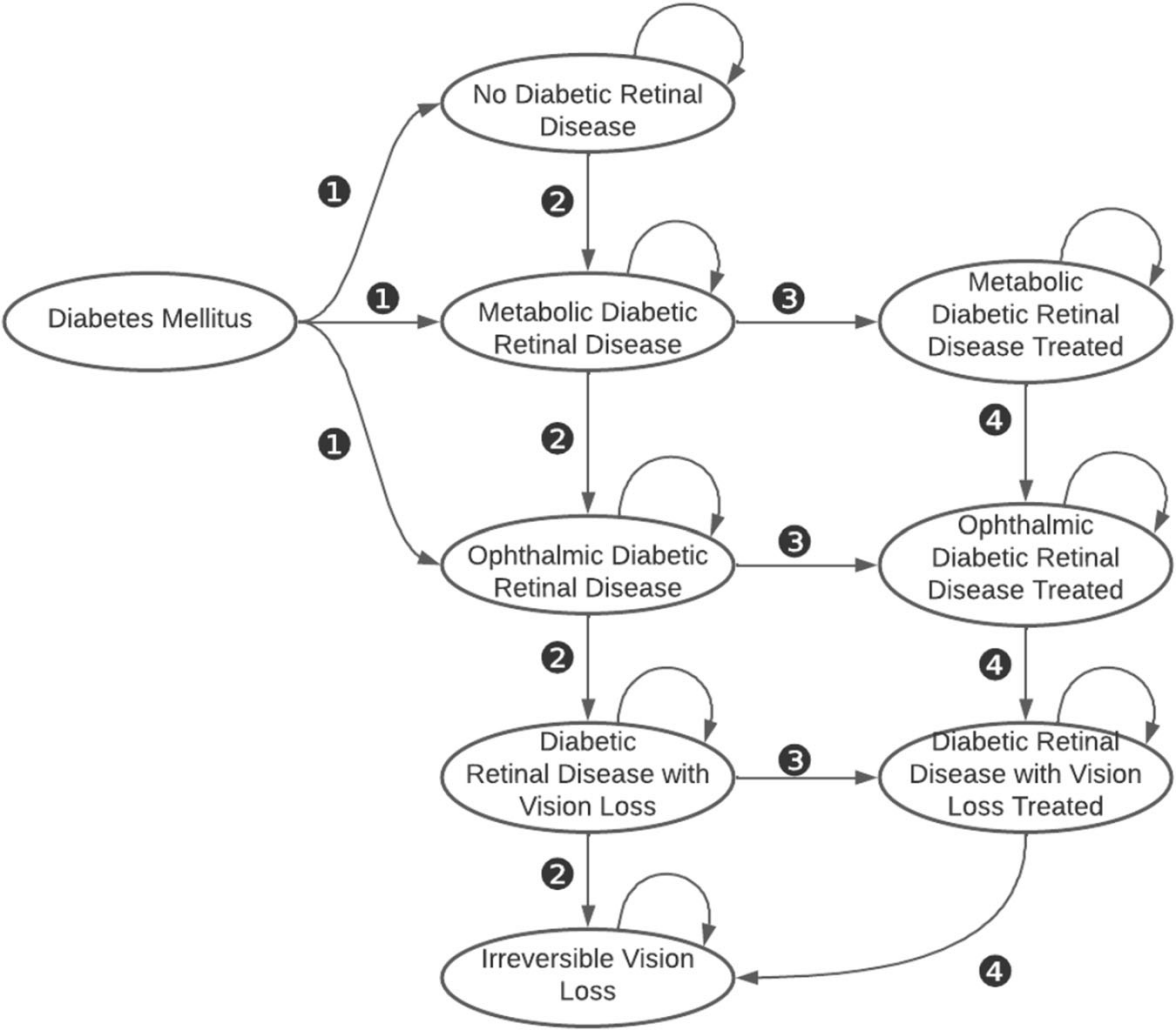
Markov model showing the states and transitions relevant to diabetic retinal disease used in the current analysis. ❶Patients with diabetes mellitus presenting to the primary care or endocrine clinic with each of the following states: No Diabetic Retinal Disease, Metabolic Diabetic Retinal Disease, or Ophthalmic Diabetic Retinal Disease. ❷Natural history transitions of diabetic retinal disease. ❸Transitions from untreated to treated diabetic retinal disease. ❹Transitions of treated diabetic retinal disease. The transitions take into account process-of-care metrics i.e., probability of accepting screening and referral in case of a positive screen, probability of disease progression, probability of adhering with recommended treatments. The structure of the Markov model is the same for both screening strategies. Table 1 shows the base-case probabilities and limits of sensitivity analysis for each parameter that are specific to the AI and ECP screening strategies. The details of the transitions specific to each strategy are represented in the decision tree in the supplement (Fig. 3 is preserved and shared on Figshare (https://figshare.com/s/ad7809b8f7010fdf83c9).

CAREVL is implemented as a computer-simulation based on a state-transition Markov model decision tree (one Markov model for each screening strategy). The model considers *population-metrics*, namely, community prevalence of the multiple severity stages of DRD, natural history of disease; *diagnostic-accuracy metrics* namely sensitivity and specificity; and *process-of-care metrics*, namely, the probability of accepting screening, of follow-up in case of a positive screen, of adherence with metabolic and ophthalmic management of DRD, and the effectiveness of metabolic and ophthalmic treatment. Figure 3 shows the states considered and the transitions between states permitted in the Markov model. The parameters of the model are probabilities and related quantities for states and transitions defined by the structure of the decision tree. The parameter values were derived from peer-reviewed published literature, and the base-case estimates are presented. Where choices for the base-case values were required, we biased the model against autonomous AI. Where relevant, the probabilities extracted from the literature were converted to transition probabilities^36^. The 12 model assumptions are detailed in the Supplementary. The models were built in TreeAge software (TreeAge Pro Healthcare version 2021 R1.1, Williamstown, MA), and we have made a spreadsheet version of the model available in the supplementary materials via Figshare (https://figshare.com/s/ad7809b8f7010fdf83c9). The parameters used in CAREVL are summarized in Table 1 and detailed in the Supplement. Fig. 3 shows the Markov-model structure used for each screening strategy. The full decision tree is in the Supplementary Fig. 1.

### Target population

The target population is adults with Type 1 or 2 DM (age > 21 years) under regular care by a primary care physician, endocrinologist or other licensed provider. The base-case assumption of prevalence of DRD and its stages in primary care was estimated from a representative sample, as this population was drawn from adult patients with diabetes presenting to primary care settings with a racial and ethnic distribution that is representative of the 37 million people with diabetes in the US^11,30,31^. People with diabetes eligible for screening were categorized into three states: no DRD, metabolic DRD or ophthalmic DRD, defined as follows: mild, moderate and severe non-proliferative DRD (ETDRS levels 35–53) primarily require metabolic control and are categorized as “metabolic DRD”; “ophthalmic DRD” is defined as DRD requiring ophthalmic treatment in addition to metabolic treatment and is taken as equivalent to ETDRS level 60 and higher (i.e., proliferative diabetic retinopathy (PDR)) or having clinically significant macular edema or center-involved macular edema, without symptoms of vision loss. The prevalences of these states were varied in sensitivity analyses. Patients with known vision loss, are recommended to go directly for eye care as opposed to first going through a screening exam and were not included in the model^37,38^.

### Screening strategies

The CAREVL model is designed and built from the patient’s perspective. Three alternative strategies for the diabetic eye exam were modeled: (1) no screening; (2) ECP strategy, where all patients are referred by the diabetes or primary care provider to an ophthalmologist or optometrist—referred to as ECP—for dilated diabetic eye exams in the clinic; (3) autonomous AI strategy, where the a digital fundus photograph is acquired and an autonomous artificial intelligence (AI) algorithm is used to analyze the image, real time result is provided, and only those diagnosed as having diabetic retinopathy or diabetic macular edema (DME) are referred for further management to ECP. The no-screening strategy was included to assess the relative impact of the other two screening strategies on expected visual outcome.

### Main outcomes and measures

The model is focused on clinical outcome—any vision loss experienced by the patient. Specifically, outcome is quantified as the probability of severe vision loss by 5 years. Because of the established benefit of treatments for PDR and DME, today, it is impossible to ethically collect natural history outcome data on untreated PDR or DME. Therefore, we used the most recent data from landmark randomized clinical trials for treatment that still had natural-history arms for PDR and DME^39,40^. In these studies, severe vision loss was defined as worse than 5/200^39^ and loss of 15 or more letters^40^ on a standardized visual acuity chart. For visual outcomes of treated DME we used data from the anti-vascular endothelial growth factor (VEGF) treated arm of diabetic retinopathy clinical research network’s protocol I^41^, a landmark clinical trial which established the effectiveness of anti-VEGF agents for DME treatment. In that study, vision loss was defined as visual acuity of 20/200 or worse. Irreversible vision loss was the probability of visual acuity of 20/200 or worse at 2 years with or without treatment in either eye.

### Sensitivity and scenario analyses

To evaluate the outcome under varying scenarios, and to account for uncertainty in base-case estimates, one-way sensitivity analyses were performed by varying one parameter at a time, while holding the others constant at their base-case estimates. Sensitivity analysis in decision analysis, to address uncertainty in model parameters, plays the same role as confidence intervals do in empirical statistical studies, to address uncertainty due to sampling. For those parameters that had different base-case values for AI vs ECP i.e., sensitivity and specificity of both strategies; accepting screening and accepting referral after screening, multiple two-way sensitivity analyses were conducted to determine if there were any scenarios where a strategy other than that identified in the base-case would be preferred. The relative impact of varying the values of key parameters on vision loss using either one of the screening strategies was evaluated. The key parameters included (1) population metrics; (2) diagnostic-accuracy metrics; (3) process-of-care metrics (see Table 1). We additionally created a series of maximal scenarios where individual parameters of process of care were set to each one’s maximum value and assessed the marginal impact of each maximum scenario over the base-case dominant strategy. The goal of the maximal-scenario analysis was to estimate the potential impact of maximizing process-of-care metrics on expected vision loss.

The manuscript complies with the Consolidated Health Economic Evaluation Reporting Standards 2022 (CHEERS) checklist^15^; this checklist was chosen because, while ours is not an economic evaluation, this checklist comes closest to governing the type of study we present.

### Reporting summary

Further information on research design is available in the Nature Research Reporting Summary linked to this article.

## Supplementary information

Supplemental Material

Reporting Summary

## Data Availability

The online supplementary material includes a macro-enabled excel sheet that provides all data that were used to build the model.

## Code availability

The model was built using the TreeAge software. No separate code was written to run the Markov model.
