# Supplementary material for: Effectiveness of artificial intelligence screening in preventing vision loss from diabetes: a policy model
Source: NPJ Digit Med. 2023 Mar 27;6:53. doi: 10.1038/s41746-023-00785-z (PMC10042864; doi:10.1038/s41746-023-00785-z)
Supplement: Supplementary file 1 — Supplemental Material [file 41746_2023_785_MOESM1_ESM.pdf]

Effectiveness of artificial intelligence compared with eye care provider screening in preventing vision loss from diabetic retinal disease: a policy model

Model Assumptions:

1. All patients in the model have diabetes (DM): they represent patients presenting to primary care or other provider managing their diabetes with no known vision loss and no diabetic retinal disease (DRD). While they might have vision loss they either do not know it or do not express it to the PCP.
2. There are three initial states: no DRD, DRD requiring metabolic control ("metabolic DRD"), DRD requiring metabolic plus ophthalmic control ("ophthalmic DRD").
3. Patients are assumed to progress from one state to the next state in the following order by single step, in other words, without skipping states. Thus, progression is step-wise from no DRD to DRD metabolic to DRD ophthalmic to DRD Vision Loss to DRD Vision Loss Irreversible. It is possible that a patient from DRD metabolic may go on to DRD Vision loss over the course of a year. However, this would lead to a higher estimate of vision loss prevented using AI. This is an example of biasing against AI.
4. Multiple years (five) are built into the same tree.
5. When a patient first presents to PCP they are always offered screening**;** if the state of DRD is known by the following year, they will not re-enter the screening pathway, but will be referred directly.
6. Our base-case probabilities are based on the Preferred Practice Pattern where available, and otherwise on the evidence from clinical trials' data where available.

7. The number of patients entering the model and dying and moving (that is, leaving the model) will be the same for all screening strategies in this time frame, and therefore we have not considered them in assessing the *difference* in effectiveness between the three alternatives
8. As the ETDRDS scale is a prognostic standard and does not encode vision loss, we have assumed that DRD metabolic and DRD ophthalmic categories do not have vision loss at the time of screening. DRD with vision loss is a separate category of patients with reversible vision loss . This vision loss may result from macular edema, vitreous hemorrhage and other manifestations of DRD.
9. Any benefit of metabolic control is not being modeled with respect to diabetes outcomes beyond DRD.
10. Any benefit of ophthalmic encounters with an ECP as opposed to AI in detecting diseases other than DRD i.e. glaucoma, AMD, cataracts is not being modeled
11. The adherence factor assumes that patients who are well controlled with regards to their A1c are following up with their PCP for metabolic control.
12. Cycle length is presumed to be 1 year because data are available for annual probabilities that intrinsically consider within-year transitions.

Uncertainty Following base-case analyses, sensitivity analyses were performed to see if the conclusions were sensitive to a wide range of probabilities.

We decided to only include data from peer-reviewed published literature for our parameters. The parameters and assumptions used to build the model are also explained in detail below.

Prevalence of Diabetic Retinopathy

The overall reported rates of DRD prevalence range from 20-50%.[1] [2-4] Mild, moderate and severe non-proliferative DRD (ETDRDS levels 35-53) require metabolic control and are categorized as **DRD Metabolic**. DRD requiring ophthalmic control **(DRDOphthalmic)** is level of DRD requiring ophthalmic treatment defined as ETDRDS level 60 and higher (i.e. proliferative diabetic retinopathy) or having clinically significant macular edema or center involved macular edema, but no known vision loss. The prevalence of DRD and its stages is estimated from the numbers in the IDx-DR pivotal trial,[3] as this population was drawn from patients with diabetes presenting to primary care settings, and is the only study with such Intent to Screen data. Frequency of macular edema in this study ranged from 2-5% depending on whether optical coherence tomography (OCT) or stereo photos were used to detect center-involved respectively clinically significant macular edema. The prevalence of DRD requiring ophthalmic control (ETDRDS 60 and higher) is estimated to be about 4% of the prevalence of DRD metabolic and is entered in the model as a formula where **propDRDOphthalmic** is 4%, the calculated value of DRD Ophthalmic in our model is 0.0088. DRD with vision loss **(prevDRDVisionLoss)** is any patient presenting to the PCP/or DM clinic who already has vision loss and will be directly referred to ECP for further evaluation without undergoing DRD screening. Prevalence of DRD with vision loss/vision impairment in a population-based cohort of patients with diabetes is estimated to range from 0.9% to 5%[5,6], if vision loss was defined as Snellen acuity of 20/200 or less the prevalence was noted to be 1.1%[5], we have therefore used a base case value of 0.01, with a range of 0 to 0.05. After the first year of screening, for follow-up years 2-5, any patient with known DRD Metabolic (**prevDRDMetabolicRx)** or

DRD Ophthalmic **(prevDRDOphthalmicRx)** does not undergo screening and is directly referred for treatment (**pDRDMetabolicReferred, pDRDOphthalmicReferred**).

Screening and Referral for Appropriate Care After Screening

The probability that a patient undergoes recommended DRD screening ranges by location and population, with ECP-based DRD screening/exam rates (**pAcceptsScreeningECP**) varying from 11-71%[2,7-11]. After a patient undergoes ECP screening, the probability of further ECP followup (**pAcceptsReferralAfterECP**) if DRD screening is positive is reported to be 29%[7]. However, this may be as high as 100% if an ophthalmologist or retina specialist is performing the DRD screening and can provide treatment at the same visit. We have accounted for this in the sensitivity analysis. When DRD screening is implemented in the outpatient diabetes care setting using point-of-care automated screening, screening rates reach 95%[12], (**pAcceptsScreeningAI**) and the likelihood of follow-up with ECP if point of care screening is positive ranges from 55-95% (**pAcceptsReferralAfterAI),** with a base case of 0.75 and a range of 0 to 0.95[7,10,13-15]. Surprisingly, overall eye care utilization rates for patients with severe and partial visual impairment are 58.3% and 49.6%, respectively, and less (35.9% and 23.8%, respectively) without health insurance. As the definition of vision loss in our paper is severe vision loss, we have used 0.58 as the base case (**pDRDVisionLossAcceptsReferral)**[16]. However, this range may be wide depending on many factors including insurance, access to care and level of vision loss, we have accounted for this in the sensitivity analysis with a range of 0 to 0.75. The parameters **pDRDMetabolicReferred** and **pDRDOphthalmicReferred** are 0 for year 1 when patients are not aware of their diagnosis and 1 for years 2-5 for

patients who have already been identified as having DRD Metabolic or DRD Ophthalmic in the previous years and are now directly referred for further care without undergoing repeat screening.

Natural history of Diabetic Retinopathy

Data on natural history of development (**pNaturalHistoryToDRDMetabolic**)

and progression of DRD metabolic in the absence of treatment, i.e. **pNaturalHistoryDRDMetabolicToOphth** and **pNaturalHistoryDRDMetabolicToVisionLoss** are extrapolated from the conventional treatment arm of the DCCT trial[17]. Data on natural history of progression of DRD Ophthalmic (i.e. PDRD and DME) to vision loss i.e. **pNaturalHistoryDRDophthToVisionLoss** are extrapolated from data from the Diabetic Retinopathy Study[18] for proliferative DRD and Early Treatment for Diabetic Retinopathy Study Report # 1 for DME[19]. Progression to severe vision loss at 5 years (defined as visual acuity of less than 5/200) among those with proliferative DRD without treatment was 33/100 eyes (0.33/eye) over 5 years for a transition rate of ln (0.77)(-5)= 0.05. The probability of untreated (clinically significant) macular edema progressing to vision loss (defined as loss of 15 or more letters) is: 24% at 3 years, for an annual rate of ln(0.75)/(-3)=0.10 [observed probability = 1-exp(-hazard rate * number of years), assuming constant hazard for each of the 3 years, so hazard = ln(1-probability)/(-number of years)]. Using the average of the two values 0.05 and 0.10, we use 0.075 for probability of natural history of progression from DRD ophthalmic to vision loss (**pNaturalHistoryDRDophthToVisionLoss**). **pNaturalHistoryVisionLossToVisionLossIrreversible** is the probability of persistent vision loss in patients with DRD who present with vision loss and do not receive treatment. We used data from the Diabetic Retinopathy Vitrectomy Study to estimate the probability of visual acuity of 20/200 or worse at 2 years in the

natural history group[20]. Given that at 2 years, 60.7% had persistent vision loss, we used the following formula (0.6 = 1 −exp(−lambda * 2)) to calculate an annual transition probability of 0.367.

Progression of Treated Diabetic Retinopathy

For progression of treated DRD Metabolic to DRD Ophthalmic (**pTreatedDRDMetabolicToOphth**), we used data from the intensive treatment arm of the DCCT[17] using the average probability of progression to proliferative retinopathy or laser treatment, we estimated an average annual progression rate from DRD metabolic to DRD ophthamic of 1%. However, real-world progression is often higher and we have accounted for this in the sensitivity analysis using an upper limit of 5%. We used data from clinical trials on treatment of DRD Ophthalmic (i.e. proliferative DRD and macular edema) to estimate the probability of progression to vision loss despite treatment of DRD requiring ophthalmic management. As pan-retinal photocoagulation (PRP) remains the standard of care for proliferative DRD, we used data from the treated arm of the Diabetic Retinopathy Study[18] to estimate probability of vision loss from proliferative DRD treated with PRP. As anti-vascular endothelial growth factor (anti-VEGF) agents have become the standard of care for center-involved DME, we used 5-year data from the landmark clinical trial conducted by the Diabetic Retinopathy Clinical Research (DRCR) network, which reported 5 year probability of severe vision loss among participants in the ranibizumab + deferred laser group (**pTreatedDRDOphthalmicToVisionLoss**), to obtain an average probability of 2% for progression to vision loss despite treatment[21]. In our sensitivity analysis we have a wide range for this (0 to 0.5) as in the real-world there are multiple factors associated with vision loss. For the probability of persistent vision loss despite treatment

(**pTreatedDRDVisionlosstoVisionLossIrreversible**), we used data from DRCR network's Protocol AB in which patients with vision impairment from vitreous hemorrhage associated with proliferative DRD were treated with anti-VEGF agent aflibercept or with pars plana vitrectomy with endolaser. At 2 years, average proportion of patients with vision loss of 20/200 or worse in the two groups was 6.8% with an annual transition probability of 3.4%[22].

Adherence Factors

Metabolic management is a key factor in affecting progression of DRD. Adherence to metabolic management of diabetes, defined as achieving lipid, blood pressure and glucose targets, ranges widely. Pantalone et al reported that 24% with Type 2 DM achieved A1c of < 8%[23]. Foster et al reported that 21 & 37% of patients with Type 1 DM achieved A1c of less than 7 & 7.5% respectively[24]. National level data shows that the percentage of adult participants with A1c of < 7% declined from 57% to 51% from 1999 to 2018 and the percentage of participants who were able to achieve lipid, blood pressure and glucose targets was only about 22%[25]. We used 0.24 as our base case for adherence to metabolic control (**pAdheringToMetabolicManagement**). Probability of adhering to ophthalmic management varies from 18-56% depending on whether treatment is recommended. We assumed that for a given individual their odds of adhering to more severe DRD would be higher than that for less severe DRD. Therefore, we divided 27% (median of 18 to 56%) by **pAdheringToMetabolicManagement** (24%) to arrive at a relative odds increased adherence to ophthalmic management (**rOddsIncAdherencetoOphthalmic**) of 0.13. The probability of adhering to ophthalmic management (**pAdheringToOphthalmicManagement)** was calculated from this odds ratio as 0.26. To estimate the probability of adhering with vision loss management (**pAdheringToVisionLossManagement**) we used data from Lee et al[16] regarding

eye care utilization rates among those with or without visual impairment. Participants with health insurance and visual impairment had eye care utilization of up to 58% (weighted probability of 50% when considering those with some and severe impairment) compared to 34% for those without visual impairment. The calculated odds increased adherence case of vision loss (**rOddsIncAdherencetoVisionLoss**) is 0.97. pAdheringToVisionLossManagement was calculated from this odds ratio as 0.41. As there is generally a wide range in adherence with recommended treatments, we have used a full range from 0 to 1 in sensitivity analyses to evaluate the impact of adherence on vision loss.

Sensitivity and Specificity of ECP and AI screening

We used data from prior literature which reports sensitivity of ECP exam for detection of referrable DRD to 33% and specificity for ECP exam to be 99%[26]. Sensitivity of AI for detection of referrable DR is reported to range from 87% to 98%, to bias against AI we have used a conservative estimate of 0.87. Specificity of AI for detection of referrable DRD is reported to be 85 to 99%, we have used a conservative estimate of 0.91 to bias the model against AI.[27-29]

Additional justification for selection of base-case values and sensitivity ranges for the three pairs of parameters that differ widely between AI and ECP

The following explanations are provided to summarize the documented notes and rationale made during the development of the model and preparation of the manuscript. While we have provided a lengthy explanation here, it is not standard to include this level of detail in a decision analysis report.

1) **Sensitivity of ECP screening vs AI screening (Base case values: ECP=0.33 vs AI=0.87; range for sensitivity analysis for both values 0 to 1)**

There is limited data regarding sensitivity of ECP exam compared to a reference standard, and specifically to the highest level reference standard according to FDA, a level I prognostic standard.[30] Furthermore, while specific clinicians may have been compared against this level of reference standard, individual clinicians have not been validated against a prognostic standard. It is not standard of care to get retinal imaging for all patients who present for a diabetes eye exam. The American Diabetes Association recommends a dilated and comprehensive eye exam by an ophthalmologist or optometrist and the American Academy of Ophthalmology recommends a regular ophthalmologic exam or screening of high-quality photographs.[31,32] We therefore considered a dilated eye exam as opposed to retinal image review by an eye care provider in determining our base case for sensitivity and the most reliable value we could find in the literature was from Pugh et al.[26] Also, there is considerable variability in the exam and access to imaging depending on whether the patient is seen by an optometrist, ophthalmologist or retina specialist. We therefore considered a broad range of 0 to 1 in our sensitivity analysis. Despite this large range in sensitivity analysis AI dominated over ECP for vision loss prevention in one-way and two-way sensitivity analyses. Sensitivity of AI detection has been 0.87 and higher in the two prospective studies completed so far that led to FDA approval of autonomous AI[27,28]. While we considered the Gulshan et al[29] study for the range in the sensitivity analysis, the number 0.87 was used as base-case and to bias against AI we used the lower of the sensitivity numbers of 0.87 (Abramoff et al)[27] and 0.96 (Ipp et al)[28] reported in the two pivotal prospective trials that have

led to FDA approval of autonomous AI. We have also used the same rationale and used similar values in our previously published decision analysis model[12].

2) **Probability of accepting screening via ECP vs AI (Base-case values: ECP=0.20 vs AI=0.95; sensitivity range for ECP: 0 to 0.8; sensitivity range AI: 0 to 1)**

The probability of accepting screening for diabetic retinopathy by an eye care provider varies widely depending on the setting. Three US-based references cited by us report ranges from 11 to 18%[2,8,10], one of which, the most extensive in > 300,000 patients, Benoit et al[2] report that 15% obtained eye screening via going to an eye care provider. We thought this number was most representative of the US population as it is based on claims data (as opposed to self-report which can be a misleading estimate for several reasons). This paper also included the largest sample size. Another paper Mansberger et al [9] reported rates of 56% for screening by an eye care provider but this was in the setting of a randomized controlled trial and in two clinics with a total of 646 patients (0.2% of the Benoit study). We tried to use nationally representative estimates as close to the real-world as possible and the reported rate by Mansberger et al is not representative of the US population and was noted in the special setting of a randomized clinical trial. Another study from An et al included ~ 200,000 participants but these were patients within an integrated health-care delivery organization.[11] They reported rates of 71% but given the special healthcare setting these are not generalizable to the US population. Also, as the authors pointed out in their paper, the rate of 71% was achieved when a generous definition of adherence

was used (adherence with annual or biennial exams). Biennial exams are only appropriate under specific situations when the patient has excellent glycemic control and there is no evidence of retinopathy for one or more annual eye exams. Based on national-level data the proportion of patients with diabetes who have lipid, BP and glucose control is 22%, hence on a national-level, annual eye exams would be most likely needed. When An et al used the annual exam guideline in their sensitivity analysis, the probability of having had screening by ECP was as low as 12%, similar to the other numbers reported in the literature.

In summary, the probability of adhering with screening by ECP has a wide reported range depending on settings and definition. We used 0.20 as the upper limit of what has been reported in the literature, biasing against AI. We also compared AI vs ECP using a wide range on sensitivity analysis of 0 to 0.8 to include all possible probabilities reported in the literature. AI dominated over ECP across this entire range.

The probability of accepting point-of-care screening by teleophthalmology or AI has been reported to be high for a patient population that is presenting for care for diabetes. Our paper using point of care AI showed that 95% of our patients accepted screening[12]. Mansberger et al reported rates of 94%[9] and Crossland et al reported rates of acceptance of point-of-care teleophthalmology screening to be 100%[7]. However, national level estimates are not yet available for AI or teleophthalmology. We, therefore used a large range of 0 to 1 in our sensitivity analysis as uptake of this technology for screening will be variable depending on the healthcare setting and access to and availability of technology.

3) **Probability of accepting referral to ECP after ECP and AI screen Base-case values: ECP= 0.29 vs AI=0.75; sensitivity range for ECP: 0 to 0.1; sensitivity range AI: 0 to 0.95)**

There are very limited data on continued follow-up with ECP after an initial positive exam by an ECP. There is also considerable variability depending on the qualifications of the ECP e.g. if an optometrist sees a patient, they would have to refer the patient to make a separate visit to see a retina specialist for treatment, whereas if a retina specialist sees a patient for their initial exam they can initiate treatment the same day and the probability of accepting referral would technically become 1.0. However, optometrist provide a large share of the initial eye exams for patients with diabetes[33,34]. Given the disconnected nature of the healthcare system where patients are often seen for follow-up eye care in a different setting from where they obtained their primary care, it is challenging to estimate the national level probability of following up after ECP or AI screening. We used data from an Australian paper, which specifically looked at this question and compared follow-up after screen positive for an eye care provider setting vs teleophthalmology setting, with a reported rate of 29% after being diagnosed with referrable disease on the initial screening by an eye care provider and 95% after teleophthalmology[7]. Other studies have reported follow-up of up to 55 to 75% after AI/teleophthalmology screening in the US. We used 0.75 as an estimate falling between the reported ranges of 55 to 95%[10,13,14,35].

Supplementary Figures and Tables

Supplementary Figure 1 shows the full decision tree model.

Supplementary Figure 2 shows the Tornado diagram

Supplementary Figure 3 shows the results of the two-way sensitivity analysis comparing probability of accepting referral after artificial intelligence (AI) versus eye-care provider (ECP) screening. The figure shows that AI is the preferred strategy except in the unlikely scenario of low probability of accepting referral after AI and a high probability of accepting referral after ECP. This scenario is far from the base-case shown by the marker (X) in the figure.

Supplementary Table 1 shows the results of various two-way sensitivity analyses.

Supplementary Figure 1 below displays the tree

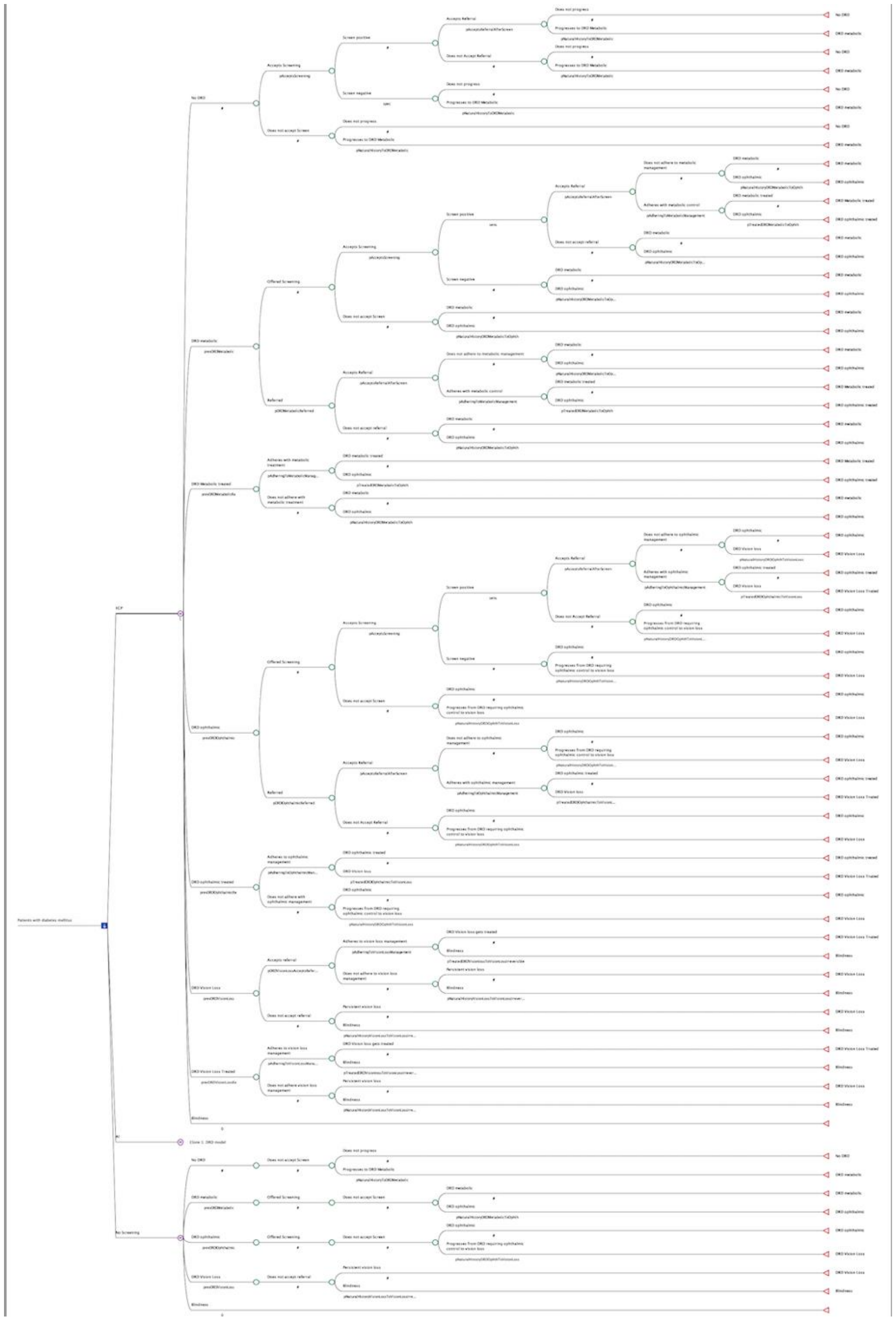

Supplementary Figure 2

https://www.DRDopbox.com/s/lboaoszll899geq/Supplementary%20Figure%201%20Markov%20all%20nodes%2003.28.2022%20all %20three%20options.png?dl=0

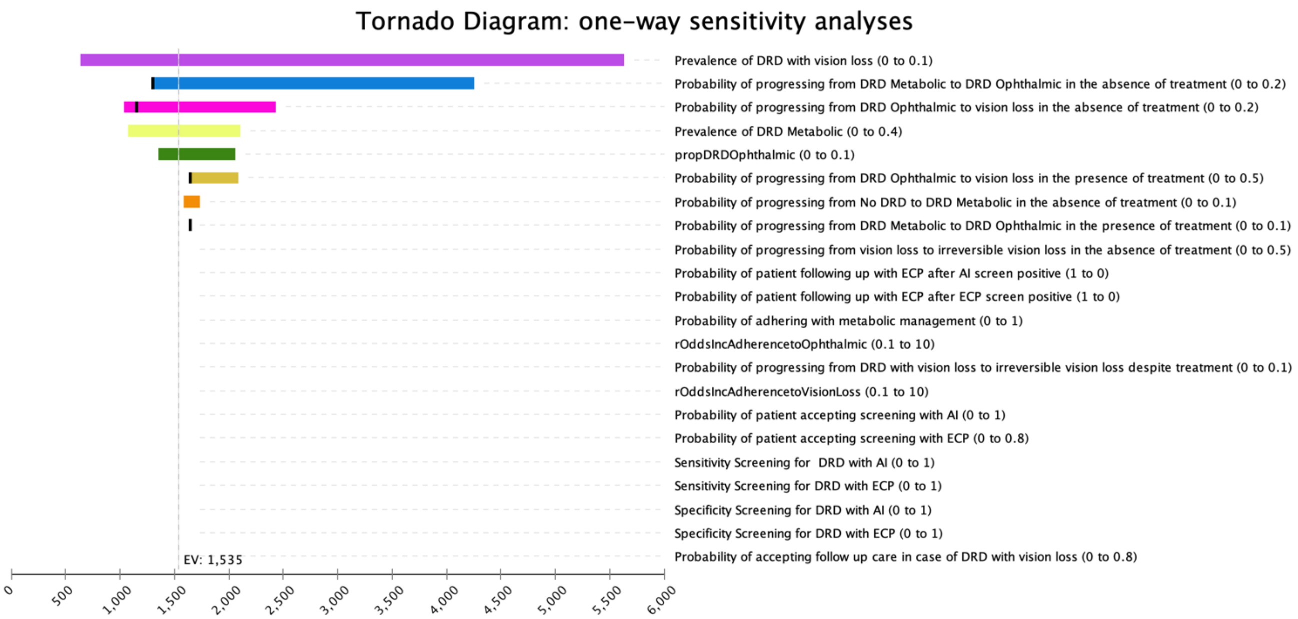

DRD=diabetic retinal disease; propDRDOphthalmic=Proportion of patients with DRD who have ophthalmic DRD: this is linked to the prevalence of DRD metabolic; rOddsIncAdherencetoOphthalmic: Odds of adhering with ophthalmic treatment relative to the probability of adhering with metabolic management; rOddsIncAdherencetoVisionLoss: odds of adhering with vision loss treatment relative to the probability of adhering with ophthalmic management;

Supplementary Figure 3

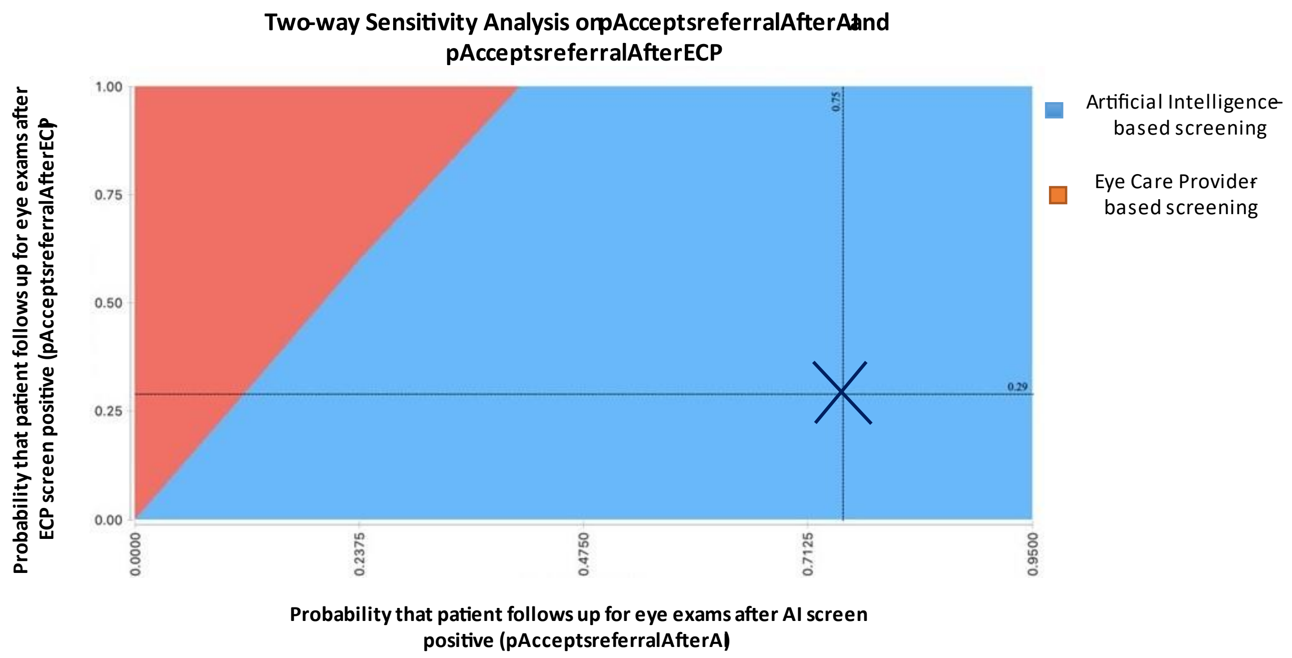

Table 1 (Supplement): Results of two-way sensitivity analyses of all parameters that were different between AI and ECP screening strategies

Parameter 1	Base case value (min, max value for sensitivity range)	Parameter 2	Base case value(min, max value for sensitivity range)	Conclusion
sensScreenAI	0.87 (0,1)	sensScreenECP	0.33 (0,1)	AI dominates
specScreenAI	0.91 (0,1)	specScreenECP	0.99 (0,1)	AI dominates
AcceptsScreenAI	0.95 (0,1)	AcceptsScreenECP	0.20 (0,0.80)	AI dominates
AcceptsReferralAfterAIScreen	0.75 (0,0.95)	AcceptsReferralAfterECP	0.29 (0,1)	AI dominates, ECP preferred in unlikely situations of low probability of AcceptsReferralAfterAIScreen and high AcceptsReferralAfterECP (output shown in supplemental figure 3)

Table 2 (Supplement): Parameters for the Decision Model

Parameter names	Parameter Description	Base-case estimate	For sensitivity analysis	
			Low	High
**Population-metrics: Prevalence and natural history of disease**				
prevDRDMetabolic	Prevalence of Metabolic DRD[27]	0.22	0	0.40
prevDRDOphthalmic	Prevalence of Ophthalmic DRD[27]	0.0088*	0	0.10
prevDRDVisionLoss	Prevalence of DRD with Vision Loss[5,6]	0.01	0	0.05
pNaturalHistoryToDRDMetabolic	No DRD to metabolic DRD[17]	0.05	0	0.15
pNaturalHistoryDRDMetabolicToOphth	Metabolic DRD to ophthalmic DRD[17]	0.02	0	0.20
pNaturalHistoryDRDophthToVisionLoss	Ophthalmic DRD to vision loss[18,19]	0.075	0	0.20
pNaturalHistoryVisionLossToVisionLossIrreversible	Vision loss to irreversible vision loss[20]	0.37	0	0.50
**Diagnostic-accuracy metrics: Sensitivity and Specificity of Screening Strategies**				
sensScreeningAI	Sensitivity of screening for DRD with AI[27-29]	0.87	0	1
sensScreeningECP	Sensitivity of screening for DRD with ECP[26]	0.33	0	1
specScreeningAI	Specificity of screening for DRD with AI[27-29]	0.91	0	1
specScreeningECP	Specificity of screening for DRD with ECP[26]	0.99	0	1
**Process-of-care metrics: Screening and Referral for Appropriate Care**				
pAcceptsReferralAfterAI	Probability that patient follows up for eye care after AI screen positive[7,10,13,14]	0.75	0	0.95
pAcceptsReferralAfterECP	Probability that patient follows up for eye exams after ECP screen positive[7]	0.29	0	1
pAcceptsScreeningAI	Probability of patient Accepting Screening by AI[12]	0.95	0	1
pAcceptsScreeningECP	Probability of patient Accepting Screening by ECP[2,7-11]	0.20	0	0.80
pDRVisionLossAcceptsReferral	Probability that patient with Vision Loss Accepts referral to ECP[16]	0.58	0	0.75
**Process-of-care metrics: Effectiveness of treatments for DRD (Progression of treated disease)**				
pTreatedDRDMetabolicToOphth	Metabolic DRD to ophthalmic DRD[17]	0.01	0	0.05
pTreatedDRDOphthalmicToVisionLoss	Ophthalmic DRD to vision loss[18,21,36]	0.02	0	0.50
pTreatedDRVisionlossToVisionLossIrreversible	Vision loss to irreversible vision loss[22]	0.034	0	0.05
	**Process-of-care metrics: Probability of Adherence to Treatment**			
pAdheringToMetabolicManagement	Adhering to metabolic management[23-25]	0.24	0	1
pAdheringToOphthalmicManagement	Adhering to ophthalmic management[16,37]	0.26*	0	1
pAdheringToVisionLossManagement	Adhering to DRD vision loss management[16]	0.41*	0	1
*Calculated values: see supplement text				

References

1. Kempen JH, O'Colmain BJ, Leske MC, et al. The prevalence of diabetic retinopathy among adults in the United States. *Archives of ophthalmology (Chicago, Ill: 1960)*. 2004;122(4):552-563.
2. Benoit SR, Swenor B, Geiss LS. Eye Care Utilization Among Insured People With Diabetes in the U.S., 2010-2014. 2019;42(3):427-433.
3. Abràmoff MD, Lavin PT, Birch M, Shah N, Folk JC. Pivotal trial of an autonomous AI-based diagnostic system for detection of diabetic retinopathy in primary care offices. *Nature Digital Medicine*. 2018;1(1):39.
4. Bursell S-E, Fonda SJ, Lewis DG, Horton MB. Prevalence of diabetic retinopathy and diabetic macular edema in a primary care-based teleophthalmology program for American Indians and Alaskan Natives. *PLoS One*. 2018;13(6):e0198551.
5. Prasad S, Kamath GG, Jones K, Clearkin LG, Phillips RP. Prevalence of blindness and visual impairment in a population of people with diabetes. *Eye (Lond)*. 2001;15(Pt 5):640-643.
6. de Fine Olivarius N, Siersma V, Almind GJ, Nielsen NV. Prevalence and progression of visual impairment in patients newly diagnosed with clinical type 2 diabetes: a 6-year follow up study. *BMC Public Health*. 2011;11:80.
7. Crossland L, Askew D, Ware R, et al. Diabetic Retinopathy Screening and Monitoring of Early Stage Disease in Australian General Practice: Tackling Preventable Blindness within a Chronic Care Model. *Journal of diabetes research*. 2016;2016:8405395.
8. Fuller SD, Hu J, Liu JC, et al. Five-Year Cost-Effectiveness Modeling of Primary Care-Based, Nonmydriatic Automated Retinal Image Analysis Screening Among Low-Income Patients with Diabetes. *Journal of Diabetes Science and Technology*. 2020:1932296820967011.
9. Mansberger SL, Gleitsmann K, Gardiner S, et al. Comparing the effectiveness of telemedicine and traditional surveillance in providing diabetic retinopathy screening examinations: a randomized controlled trial. *Telemedicine and e-Health*. 2013;19(12):942-948.
10. Liu J, Gibson E, Ramchal S, et al. Diabetic Retinopathy Screening with Automated Retinal Image Analysis in a Primary Care Setting Improves Adherence to Ophthalmic Care. *Ophthalmology Retina*. 2020.
11. An J, Niu F, Turpcu A, Rajput Y, Cheetham TC. Adherence to the American Diabetes Association retinal screening guidelines for population with diabetes in the United States. *Ophthalmic epidemiology*. 2018;25(3):257-265.
12. Wolf RM, Liu TA, Thomas C, et al. The SEE Study: Safety, Efficacy, and Equity of Implementing Autonomous Artificial Intelligence for Diagnosing Diabetic Retinopathy in Youth. *Diabetes care*. 2021;44(3):781-787.
13. Jani PD, Forbes L, Choudhury A, Preisser JS, Viera AJ, Garg S. Evaluation of Diabetic Retinal Screening and Factors for Ophthalmology Referral in a Telemedicine Network. *JAMA Ophthalmology*. 2017;135(7):706-714.

14. Stebbins K, Kieltyka S, Chaum E. Follow-Up Compliance for Patients Diagnosed with Diabetic Retinopathy After Teleretinal Imaging in Primary Care. *Telemedicine and e-Health.* 2021;27(3):303-307.
15. Wolf RM, Liu TYA, Thomas C, et al. The SEE Study: Safety, Efficacy, and Equity of Implementing Autonomous Artificial Intelligence for Diagnosing Diabetic Retinopathy in Youth. *Diabetes Care.* 2021;44(3):781-787.
16. Lee DJ, Lam BL, Arora S, et al. Reported Eye Care Utilization and Health Insurance Status Among US Adults. *Arch Ophthalmol-Chic.* 2009;127(3):303-310.
17. Group TDCaCTR. The effect of intensive treatment of diabetes on the development and progression of long-term complications in insulin-dependent diabetes mellitus. *New Engl J Med.* 1993;329(14):977-986.
18. Diabetic Retinopathy Study G. Photocoagulation treatment of proliferative diabetic retinopathy: clinical application of DRS findings: DRS report 8. *Ophthalmology.* 1981;88:583-600.
19. Photocoagulation for Diabetic Macular Edema: Early Treatment Diabetic Retinopathy Study Report Number 1 Early Treatment Diabetic Retinopathy Study Research Group. *Arch Ophthalmol-Chic.* 1985;103(12):1796-1806.
20. Group DRVSR. Early vitrectomy for severe vitreous hemorrhage in diabetic retinopathy. Two-year results of rancomized trial, Diabetic retinopathy vitrectomy study report 2. *Arch Ophthalmol.* 1985;103:1644-1652.
21. Elman MJ, Ayala A, Bressler NM, et al. Intravitreal Ranibizumab for diabetic macular edema with prompt versus deferred laser treatment: 5-year randomized trial results. *Ophthalmology.* 2015;122(2):375-381.
22. Antoszyk AN, Glassman AR, Beaulieu WT, et al. Effect of intravitreous aflibercept vs vitrectomy with panretinal photocoagulation on visual acuity in patients with vitreous hemorrhage from proliferative diabetic retinopathy: a randomized clinical trial. *Jama.* 2020;324(23):2383-2395.
23. Pantalone KM, Misra-Hebert AD, Hobbs TM, et al. The Probability of A1C Goal Attainment in Patients With Uncontrolled Type 2 Diabetes in a Large Integrated Delivery System: A Prediction Model. *Diabetes Care.* 2020;43(8):1910-1919.
24. Foster NC, Beck RW, Miller KM, et al. State of Type 1 Diabetes Management and Outcomes from the T1D Exchange in 2016-2018. *Diabetes Technol Ther.* 2019;21(2):66-72.
25. Fang M, Wang D, Coresh J, Selvin E. Trends in diabetes treatment and control in US adults, 1999–2018. *New Engl J Med.* 2021;384(23):2219-2228.
26. Pugh JA, Jacobson JM, Van Heuven W, et al. Screening for diabetic retinopathy: the wide-angle retinal camera. *Diabetes care.* 1993;16(6):889-895.
27. Abràmoff MD, Lavin PT, Birch M, Shah N, Folk JC. Pivotal trial of an autonomous AI-based diagnostic system for detection of diabetic retinopathy in primary care offices. *npj Digital Medicine.* 2018;1(1):39.
28. Ipp E, Liljenquist D, Bode B, et al. Pivotal Evaluation of an Artificial Intelligence System for Autonomous Detection of Referrable and Vision-Threatening Diabetic Retinopathy. *JAMA network open.* 2021;4(11):e2134254-e2134254.
29. Gulshan V, Peng L, Coram M, et al. Development and validation of a deep learning algorithm for detection of diabetic retinopathy in retinal fundus photographs. *Jama.* 2016;316(22):2402-2410.
30. Channa R, Wolf R, Abramoff MD. Autonomous artificial intelligence in diabetic retinopathy: from algorithm to clinical application. *Journal of diabetes science and technology.* 2021;15(3):695-698.

31. Association AD. 11. Microvascular complications and foot care: Standards of Medical Care in Diabetes—2021. *Diabetes Care*. 2021;44(Supplement_1):S151-S167.
32. Flaxel CJ, Adelman RA, Bailey ST, et al. Diabetic Retinopathy Preferred Practice Pattern®. *Ophthalmology*. 2020;127(1):P66-p145.
33. Olsen CL, Gerber TM, Kassoff A. Care of diabetic patients by optometrists in New York State. *Diabetes care*. 1991;14(1):34-41.
34. Tang V, Symons RCA, Guest D, McKendrick AM. An overview of optometrists' diabetic retinopathy practice patterns–a cross-sectional survey. *Ophthalmic and Physiological Optics*. 2021;41(4):885-895.
35. Mathenge W, Whitestone N, Nkurikiye J, et al. Impact of Artificial Intelligence Assessment of Diabetic Retinopathy on Referral Service Uptake in a Low-Resource Setting: The RAIDERS Randomized Trial. *Ophthalmology Science*. 2022;2(4):100168.
36. Wykoff CC, Khurana RN, Nguyen QD, et al. Risk of Blindness Among Patients With Diabetes and Newly Diagnosed Diabetic Retinopathy. *Diabetes Care*. 2021;44(3):748-756.
37. Bresnick G, Cuadros JA, Khan M, et al. Adherence to ophthalmology referral, treatment and follow-up after diabetic retinopathy screening in the primary care setting. *BMJ Open Diabetes Res Care*. 2020;8(1).
